# Draft genome sequences of related *Paeniglutamicibacter* sp. isolates from two disparate cave systems

**DOI:** 10.1128/mra.00127-25

**Published:** 2025-04-29

**Authors:** Earl Middlebrook, Jennifer Hathaway, Anastasia Pittis, George Abernathy, Karen Davenport, Cheryl Gleasner, Kimberly Mcmurry, Julia Kelliher, Andrew W. Bartlow, Migun Shakya, Michaeline Albright, Anand Kumar, Blake Hovde, Brett Youtsey, Buck Hanson, Aaron Robinson, Mark Flynn, Shannon Johnson, Diana Northup, Armand E. K. Dichosa

**Affiliations:** 1 B-GEN: Genomics and Bioanalytics, Bioscience Division, Los Alamos National Laboratory 5112, Los Alamos, New Mexico, USA; 2 Department of Biology, The University of New Mexico 118833, Albuquerque, New Mexico, USA; 3 B-TEK: Biochemistry and Biotechnology, Bioscience Division, Los Alamos National Laboratory 5112, Los Alamos, New Mexico, USA; 4 Allonnia, Boston, Massachusetts, USA; 5 B-IOME: Microbial and Biome Sciences, Bioscience Division, Los Alamos National Laboratory 5112, Los Alamos, New Mexico, USA; DOE Joint Genome Institute, Berkeley, California, USA

**Keywords:** cave, draft genome, bacterial isolates, 16S sequencing, whole genome sequencing, bioinformatics

## Abstract

We present the genome assemblies of two similar *Paeniglutamicibacter* strains, ORCA_105 and MACA_103, isolated from Mammoth and Oregon Cave systems, respectively. These closely related, but distinct genomes will provide a resource for those studying genomic adaptation to caves.

## ANNOUNCEMENT

Many bacterial lineages thrive in extreme terrestrial cave systems (1, 2) and have yet to be identified. Here, we present draft genomes of two *Paeniglutamicibacter* isolates from Oregon and Kentucky caves with identical 16S rRNA genes.

Isolate ORCA_105 was collected on 3 October 2018 from the floor of Oregon Caves National Monument (ORCA) 68 m from the cave entrance (Oregon, USA; UTM NAD83 10T 466241, 4661543) using a sterile swab and inoculated onto a ½ R2A agar plate with nystatin (150,000 U/L). Isolate MACA_103 was collected on 9 April 2019 from Mammoth Cave National Park (MACA) ceiling (Kentucky, USA; UTM NAD83 16S 581551, 4109203) 400 m from the cave entrance and inoculated onto ⅕ R2A agar with 5 g/L of pulverized cave rocks and nystatin (150,000 U/L). Isolates with unique morphologies were subcultured using streak isolation and incubation at 8°C until the isolate appeared pure, at which time DNA extraction was performed from the lawn using a 1.0 µL loop.

DNA extractions were performed using the Biospec BeadBeater (Bartlesville, OK) for 1.5 min at medium speed and DNeasy UltraClean Microbial Kit (Qiagen Germantown, MD). Genomic libraries were generated with NEBNext Ultra DNA II Library Kit (New England Biolabs, Cat. #E7645L) and sequenced on an Illumina NextSeq 500 with the Mid Output Kit v2.5 (Illumina, Cat. #20024905) generating 2 × 151 bp reads. Sequence analysis was performed with the EDGE bioinformatics UI platform (v2.4.0) (3). Reads were first trimmed/filtered using *faQC* (v2.08) (4) with three bases clipped from each end and removing reads below 20 average quality and/or 50 bp length, then assembled using *IDBA* (v1.1.1) (5) with options “--pre_correction --mink 31—maxk 121—step 20 min_contig 200.” Contigs with less than 100× coverage, identified by *BWA* (v0.7.12) (6), were removed. *CheckM* (v1.2.2) (7) was used to estimate completeness and contamination, whereas *Prokka* (v1.14.5) (8) was used to predict CDSs, tRNAs, and rRNAs. Assembly details are provided in Table 1.

**TABLE 1:** Genome assembly statistics, BLAST results, and accession numbers

Characteristic	ORCA_105	MACA_103
No. of reads	32,351,542	32,156,656
Assembly size (MBP)	4.42	4.80
No. of contigs	118	147
N50 values (BP)	89,925	88,696
Max contig len	237,673	202,090
Avg. coverage	1062	979
GC content (%)	0.64968	0.65791
Best hit taxon	*P. sulfureus* and *P. antarcticus*	*P. sulfureus*
Best hit accn.	NR_026237.1 and NR_115079.1	NR_026237.1
Est. complete (%)	99.77	99.54
Est. contamination (%)	1.87	2.29
No. coding sequences^*a*^	3,934	4,257
No. rRNA^*a*^	7	4
No. tRNA^*a*^	62	62
GenBank assembly accn.*^b^*	GCF_045798545.1	GCF_045798555.1
NCBI BioSample accn.	SAMN44524352	SAMN44524351
NCBI SRA accn.	SRX27112100	SRX27112101

^*a*^ Annotations provided by Prokka (v1.14.5) (8).

^*b*^ Annotations of GenBank entries were provided by PGAP (12).

**Fig 1:**
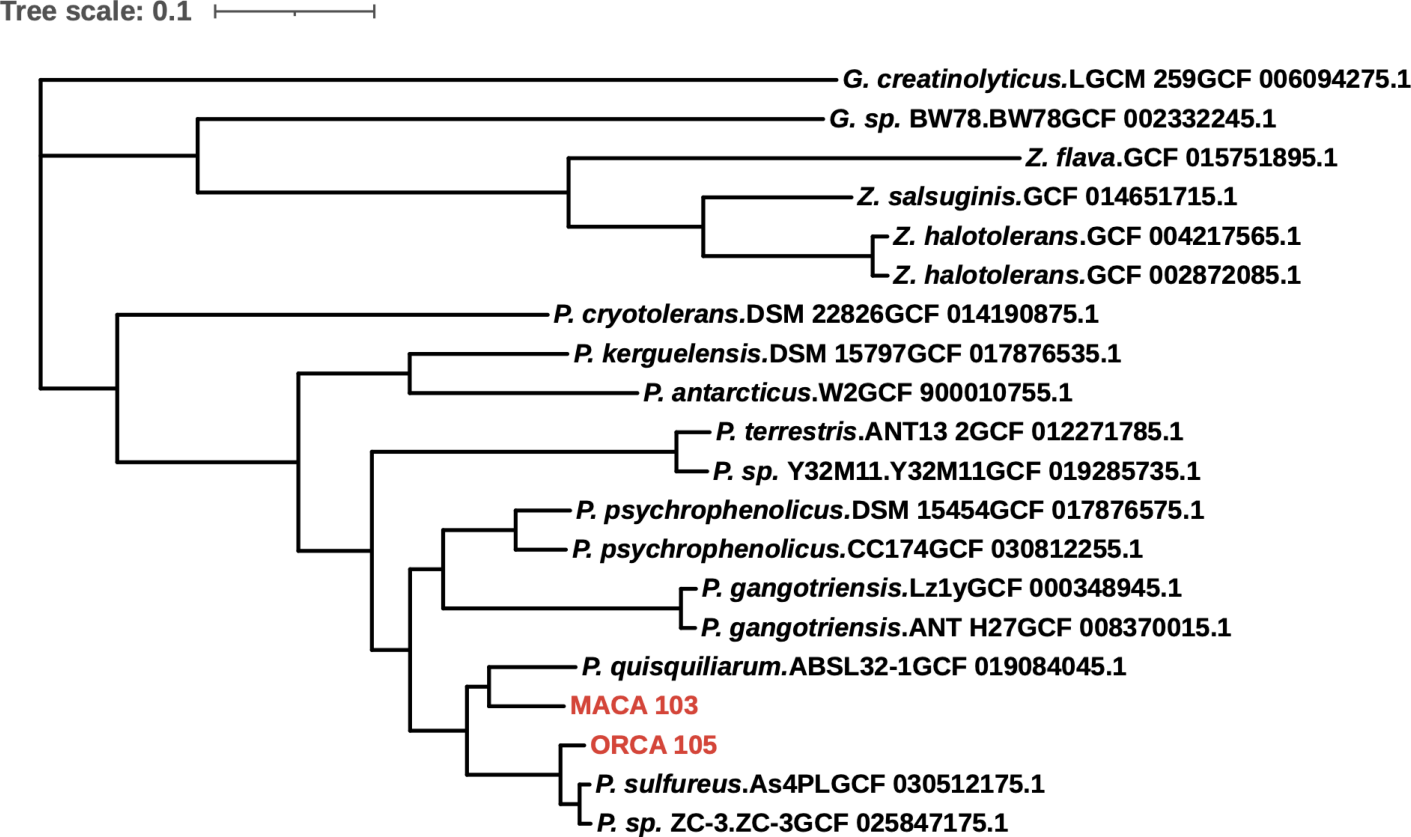
Phylogenomic tree of ORCA_105 and MACA_103 along with 18 closely related isolates. The tree was inferred with the *OrthoPhyl* pipeline (v1.0) (12). Briefly, *OrthoFinder* (v2.5.4) (13, 14) was used to infer 1,122 strict single-copy orthologs that were converted to codon alignments with *PAL2NAL* (15). *IQ-TREE* (v2.2.0.3) (16–18) was used to infer the final tree from the concatenated alignments. The tree was visualized with iTOL (19). Only bootstrap supports (calculated from 100 replicates) that are less than 100 are shown on the tree. NCBI accessions are shown at leaves. G: *Glutamicibacter*, *P*: *Paeniglutamicibacter,* and Z: *Zhihengliuella.*

The end-trimmed alignment of ORCA_105 and MACA_103 16S rRNA gene sequences shows that they are identical. By BLAST search (9–11), full-length ORCA_105 and MACA_103 16S gene sequences show very high similarity to the 16S rRNA gene of *Paeniglutamicibacter sulfureus* (NR_026237.1) at 99.47% and 99.85%, respectively, and *P. antarcticus* (NR_115079.1) at 99.47% and 99.70%, respectively. Using CEANIA (https://github.com/eamiddlebrook/CEANIA), these two strains have an average nucleotide identity of 0.903 across their core genes. A phylogenomic tree with related bacteria shows a close association between ORCA_105 and MACA_103 and *P. sulfureus* As4PL and *P. quisquiliarum* ABSL32-1, respectively (Fig. 1).

These assemblies from cave-adapted *Paeniglutamicibacter* strains will be valuable additions to the growing number of genomic sequences from cave environments for identifying genomic signatures of cave adaptation.

## Data Availability

These whole-genome projects have been deposited in NCBI’s GenBank under the BioProject PRJNA1180589 and BioSample accessions SAMN44524352 (ORCA_105) and SAMN44524351 (MACA_103). Raw reads have been deposited in NCBI’s SRA with accessions SRX27112100 (ORCA_105) and SRX27112101 (MACA_103). Assemblies are available with accessions GCF_045798545.1 (ORCA_105) and GCF_045798555.1 (MACA_103).

## References

[1] Lange-Enyedi NT, Németh P, Borsodi AK, Spötl C, Makk J. 2024. Calcium carbonate precipitating extremophilic bacteria in an alpine ice cave. Sci Rep 14:2710. doi:10.1038/s41598-024-53131-y 38302670 PMC10834452

[2] Kosznik-Kwaśnicka K, Golec P, Jaroszewicz W, Lubomska D, Piechowicz L. 2022. Into the unknown: microbial communities in caves, their role, and potential use. Microorganisms 10:222. doi:10.3390/microorganisms10020222 35208677 PMC8877592

[3] Li P-E, Lo C-C, Anderson JJ, Davenport KW, Bishop-Lilly KA, Xu Y, Ahmed S, Feng S, Mokashi VP, Chain PSG. 2017. Enabling the democratization of the genomics revolution with a fully integrated web-based bioinformatics platform. Nucleic Acids Res 45:67–80. doi:10.1093/nar/gkw1027 27899609 PMC5224473

[4] Lo C-C, Chain PSG. 2014. Rapid evaluation and quality control of next generation sequencing data with FaQCs. BMC Bioinformatics 15:366. doi:10.1186/s12859-014-0366-2 25408143 PMC4246454

[5] Peng Y, Leung HCM, Yiu SM, Chin FYL. 2012. IDBA-UD: a de novo assembler for single-cell and metagenomic sequencing data with highly uneven depth. Bioinformatics 28:1420–1428. doi:10.1093/bioinformatics/bts174 22495754

[6] Li H. 2013. Aligning sequence reads, clone sequences and assembly contigs with BWA-MEM. arXiv 1303:3997. 10.48550/arXiv.1303.3997.

[7] Parks DH, Imelfort M, Skennerton CT, Hugenholtz P, Tyson GW. 2015. CheckM: assessing the quality of microbial genomes recovered from isolates, single cells, and metagenomes. Genome Res 25:1043–1055. doi:10.1101/gr.186072.114 25977477 PMC4484387

[8] Seemann T. 2014. Prokka: rapid prokaryotic genome annotation. Bioinformatics 30:2068–2069. doi:10.1093/bioinformatics/btu153 24642063

[9] Altschul SF, Gish W, Miller W, Myers EW, Lipman DJ. 1990. Basic local alignment search tool. J Mol Biol 215:403–410. doi:10.1016/S0022-2836(05)80360-2 2231712

[10] Zhang Z, Schwartz S, Wagner L, Miller W. 2000. A greedy algorithm for aligning DNA sequences. J Comput Biol 7:203–214. doi:10.1089/10665270050081478 10890397

[11] Morgulis A, Coulouris G, Raytselis Y, Madden TL, Agarwala R, Schäffer AA. 2008. Database indexing for production MegaBLAST searches. Bioinformatics 24:1757–1764. doi:10.1093/bioinformatics/btn322 18567917 PMC2696921

[12] Middlebrook EA, Katani R, Fair JM. 2023. OrthoPhyl – a turn-key solution for large scale whole genome bacterial phylogenomics. bioRxiv. 10.1101/2023.06.27.546815.

[13] Emms DM, Kelly S. 2015. OrthoFinder: solving fundamental biases in whole genome comparisons dramatically improves orthogroup inference accuracy. Genome Biol 16:157. doi:10.1186/s13059-015-0721-2 26243257 PMC4531804

[14] Emms DM, Kelly S. 2019. OrthoFinder: phylogenetic orthology inference for comparative genomics. Genome Biol 20:238. doi:10.1186/s13059-019-1832-y 31727128 PMC6857279

[15] Suyama M, Torrents D, Bork P. 2006. PAL2NAL: robust conversion of protein sequence alignments into the corresponding codon alignments. Nucleic Acids Res 34:W609–12. doi:10.1093/nar/gkl315 16845082 PMC1538804

[16] Nguyen L-T, Schmidt HA, von Haeseler A, Minh BQ. 2015. IQ-TREE: a fast and effective stochastic algorithm for estimating maximum-likelihood phylogenies. Mol Biol Evol 32:268–274. doi:10.1093/molbev/msu300 25371430 PMC4271533

[17] Kalyaanamoorthy S, Minh BQ, Wong TKF, von Haeseler A, Jermiin LS. 2017. ModelFinder: fast model selection for accurate phylogenetic estimates. Nat Methods 14:587–589. doi:10.1038/nmeth.4285 28481363 PMC5453245

[18] Hoang DT, Chernomor O, von Haeseler A, Minh BQ, Vinh LS. 2018. UFBoot2: improving the ultrafast bootstrap approximation. Mol Biol Evol 35:518–522. doi:10.1093/molbev/msx281 29077904 PMC5850222

[19] Letunic I, Bork P. 2021. Interactive Tree Of Life (iTOL) v5: an online tool for phylogenetic tree display and annotation. Nucleic Acids Res 49:W293–W296. doi:10.1093/nar/gkab301 33885785 PMC8265157

